# Modulation of flash ERGs by dynamic backgrounds

**DOI:** 10.1007/s10633-022-09902-x

**Published:** 2022-10-20

**Authors:** Jan Kremers, Avinash J. Aher, Cord Huchzermeyer

**Affiliations:** Experimental Ophthalmology, Section for Retinal Physiology, University Hospital Erlangen, Schwabachanlage 6, 91054 Erlangen, Germany

**Keywords:** Electroretinogram, Human, Pulse stimuli, Sine wave, Weber fraction

## Abstract

**Purpose:**

The aim of this study was to characterize the signal processing mechanisms that lead to an ERG response and to use this characterization for obtaining more robust responses in patients who display feeble responses with standard recordings. We studied the influence of sinusoidally modulating backgrounds on flash ERGs and the relationship between the ERG components’ amplitudes and the momentary Weber fraction of the flash stimulus.

**Methods:**

ERG recordings were performed in nine healthy subjects and three RP patients. In four normal subjects, we measured the response to flashes (500 cd/m^2^, 1 ms duration) on a steady background (50 cd/m^2^) and on a sine wave (50 cd/m^2^ mean luminance) modulating background at 1, 5, 10, and 25 Hz temporal frequencies. The flashes were delivered at eight different phases (0–315° in a step of 45°) during the modulating background sine wave. The responses to the backgrounds were also recorded and subtracted from the responses to flash plus modulating backgrounds to obtain the flash ERGs at the different phases. The recordings in the remaining five normal subjects and the RP patients were performed with a subset of these stimuli.

**Results:**

The flash ERGs were strongly modulated by the backgrounds particularly at low frequencies and were enhanced when the momentary Weber fraction was large. The amplitudes of the components could be described by the Weber fraction plus a saturating nonlinearity and a delay in the processing of background luminance. The strength of the modulation decreased with increasing peak time of the component. Furthermore the background luminance delay was positively correlated with the peak time. The effect was also present in RP patients.

**Conclusions:**

A sine wave background of about 1 Hz can be used to enhance ERG responses. Weber fraction of the flashes is an adequate quantification of stimulus for describing the amplitudes of the ERGs. The data provide basic information on how background luminance is processed in ERG generating mechanisms. The response enhancement can be used in clinical applications to obtain a more robust comparison between normal and patient data.

## Introduction

The flash is arguably the most widely employed stimulus to elicit electroretinographical (ERG) responses. The flash ERG is standardized by the International Society for Clinical Electrophysiology of Vision (ISCEV) to ensure comparability of the results obtained at different institutions and in different experiments [1]. The flashes are generally presented upon a static background that determines the state of adaptation. The influence of the state of adaptation can then be studied by using different background luminances. Flashes on static backgrounds with variable luminances are also used in psychophysical experiments. Before the ERG recordings or psychophysical measurements commence, the static backgrounds are displayed for a sufficient amount of time to reach a steady state of adaptation. In addition, the time between flashes should be long enough to avoid that they influence the state of adaptation.

The strengths of flash stimuli in electrophysiological and psychophysical experiments is generally quantified by the Weber fraction which is the flash luminance normalized to the background luminance (I_flash_/I_bkgnd_) (see [2] for a review). There are ranges of background intensities where stimuli with equal Weber fractions elicit equal responses or sensitivities (Weber’s law). Deviations from Weber’s law are found at low and high background intensities. At low background intensities, the electrophysiological amplitudes and psychophysical sensitivities are determined by the flash intensity without normalization (linear range) or with partial normalization (de Vries–Rose law) to the background (sometimes this whole dependency range is described as Weber’s law range [2]). At high background luminance levels, saturation mechanisms are active. As a result, in the two ranges the response amplitudes and the sensitivities are smaller than expected based on Weber’s law.

An alternative manner to study the influence of the Weber fraction on ERG responses may be by using modulating (i.e. dynamic) backgrounds and by presenting the flashes at different instances during the background modulation. This results in continuous variations in Weber fraction. If the background modulation is fast, then adaptation processes can be neglected. Adaptation processes typically have time constants in the order of seconds or longer. Therefore, it can be expected that sinusoidally modulating backgrounds with temporal frequencies of 1 Hz and higher will not alter the state of adaptation. To our knowledge, dynamic backgrounds have not been used before in flash ERG measurements. However, the paradigm has been used in psychophysical studies [3] and physiological recordings from primate horizontal cells [4, 5]. The data suggest that fast processes (for the physiological recordings) and processing in different post-receptoral pathways (for the psychophysical data) may be involved. The psychophysical data also show that thresholds are smallest when the luminance of the modulating background was minimal.

It is the purpose of the present study to describe the dependency of the amplitudes and peak times of different flash ERG components as a function of the phase of the flash relative to a sinusoidally modulating background and to obtain insights in basic retinal signal processing in ERG generating pathways. By using this approach, we wanted to study if Weber fraction is an adequate quantification of stimulus strength and to model the measured responses by including additional physiological processes such as saturation. We further studied the influence of the temporal frequency of the modulating background on the flash ERGs. It can be expected that the temporal frequency of the modulating background has an influence because, at temporal frequencies that are too high to be resolved, the resultant flash ERGs should be identical to those upon a steady background. We also studied if the flash might have a reversed effect on the response to the modulating background. By describing the response changes in the different flash ERG components, it then would be possible to characterize the basic properties of their underlying mechanisms.

We compared the results in normal subjects with those of RP patients, in order to establish if the use of the flash ERGs on modulating background may have advantages in clinical applications.

## Methods

### Subjects

Nine healthy trichromats (four females; age between 24 and 61 years) and three female RP patients (age between 31 and 50 years) participated in the present study. The normal subjects received a full ophthalmological investigation and had normal color vision as established with an anomaloscope and with ERG recordings of cone opponent mechanisms [6–8].

We also measured three patients. One RP patient had a best-corrected visual acuity of decimal 0.4 (20/50) in the right eye (− 2.5 sph − 2.5 cyl/110°). She had undergone radial keratotomy for high myopia at age 18, and she had been diagnosed with RP 5 years later. Her visual field showed marked constriction in the upper hemifield and a ring-shaped scotoma in the lower hemifield. Funduscopy revealed myopic fundi with tilted disc and peripheral bone spicules. A typical hyperfluorescent ring was found in the fundus autofluorescence images. The ERG, which is described in detail in the Results section, and the standard flash ERGs [1] supported the diagnosis of RP, and this diagnosis was then genetically confirmed.

Congenital color vision defects were ruled out using the anomaloscope and the Cambridge Color Test (CCT, trivector test, implemented on the Metropsis System, Cambridge Research Systems, Cambridge, UK). The anomaloscope showed a slight shift toward red (Anomal quotient -AQ- between 0.73 and 0.83), which is frequently seen in retinal dystrophies (pseudoprotanomaly), but the CCT results were within normal limits.

The two other patients were heterozygous female carriers of X-chromosomal RPGR mutations with clinically manifest signs of rod-cone dystrophy [9]. They were 31 and 49 years old and had nyctalopia, moderate constriction of visual fields, reduced visual acuity and dyschromatopsia. Visual acuities in the study eyes were 0.32 decimal (20/62; −10.25 sph −1 cyl/5°) in the younger, and 0.4 decimal (20/50; −2.0 sph −0.5 cyl/43°) in the older patient. Fundus autofluorescence revealed a pathognomonic radial pattern [10], but pigmentary changes and disruption of the ellipsoid layer and the outer limiting membrane in the OCT were sparse (F-pattern) [9]. The older of these two patients also has diabetes mellitus, but no signs of diabetic retinopathy on funduscopy. Standard flash ERG amplitudes were markedly reduced but above noise level.

In the CCT, the younger patient had severely increased thresholds along all three axes (protan, deutan, and tritan) and a large matching range in the examination with the anomaloscope. However, the matching range included an AQ of 1, arguing against X-chromosomal color vision defects. The older RPGR carrier had only slightly elevated thresholds in the CCT examination. Like the first patient, she also displayed a slight shift towards red in the anomaloscope examination (AQ of 0.83).

The subjects were informed and signed a written consent before ERG measurements. The study followed the tenets of the Declaration of Helsinki and was approved by the ethics committee of the Friedrich-Alexander University Erlangen-Nürnberg.

### ERGs measurements

ERG recordings were performed on the right eyes of the subjects using the RETIport system (Roland Consult GmbH, Brandenburg, Germany). The eyes were dilated with a drop of 0.5% tropicamide (Stulln Pharma GmbH, Germany). The left eyes were occluded using an eyepatch during the recordings. A commercially available fiber electrode (Roland Consult GmbH, Germany), placed over the lower conjunctiva and attached to the inner and outer canthus, served as the active electrode. The skin on the forehead and the ipsilateral temple were cleaned with NuPrep® abrasive gel (D.O. Weaver & Co., Colorado, USA) and gold cup electrodes, filled with electrode paste (DO weaver & Co.), were attached with adhesive tape. They served as ground (forehead) and reference (ipsilateral temple). The impedance of the electrodes was below 5 KΩ. The signals were amplified 100,000 times, band-pass filtered between 0.3 and 300 Hz, and sampled at 2048 Hz. The ERG responses from 80 sweeps (two trials of 40 sweeps), each lasting 1 s, were averaged. To avoid onset artifacts, the first 2 s of recording time after start of the stimulation were discarded.

### Visual stimuli

The visual stimuli were displayed using a Ganzfeld stimulator (Q450SC; Roland Consult GmbH, Brandenburg, Germany) with six different LED arrays as primaries. The visual stimuli were created through CSV files that were imported by the RetiPort system (Roland Consult) to determine the output (in cd/m^2^) of each LED array in one millisecond steps. The output of the LEDs were checked using a Minolta LS-110 photometer. In the current experiments, only the white LEDs (CIE coordinates: x: 0.3157; y: 0.3327) were activated and were used to create isochromatic luminance stimuli. Three stimulus types were employed:

[A]: A 500 cd/m^2^ 1 ms (i.e. 0.5 cd.s/m^2^) flash upon a steady 50 cd/m^2^ background. One flash was presented per sweep (and thus the inter-flash interval was 1 s).
[B]: A luminance sine wave with 100% Michelson contrast and 50 cd/m^2^ mean luminance. One sweep lasted 1 s. The sine wave stimuli had 1, 5, 10 and 25 Hz temporal frequencies.
[C]: 500 cd/m^2^ 1 ms flashes upon sine wave background stimuli identical to those described in [B]. The flashes were presented at eight different phases during the sine wave background between 0 and 315° in 45° steps. The momentary luminances of the sinusoidal stimulus at 0 and 180° equaled the mean luminance and that of the steady background in [A] (i.e. 50 cd/m^2^). The luminance of the sine wave was maximal at 90° and equaled twice the mean luminance (i.e. 100 cd/m^2^). Its luminance was 0 cd/m^2^ at 270°. When using 5, 10 and 25 Hz modulating backgrounds, the flashes were displayed once in a 1 s sweep and only during the first sine wave period. Thus, the time-averaged luminance was equal for all stimuli that contained flashes [C].

With four normal subject, all background frequencies and all phases of the flashes were measured. In the remaining five normal subjects, only 1, 5 and 10 Hz backgrounds were used and the responses to the combined stimuli with the flashes at phases, 0, 90, 225, 270 and 315° were measured. Responses to the sine wave backgrounds and to the flashes on a steady background were also measured. Measurements for one background frequency were completed before measuring another frequency. The flashes at different phases were presented randomly. In addition, the sequence of background frequencies was randomized.

With the RP patients, we measured the responses to a flash upon a steady background (as described in [A]), to a 1 Hz 100% sine-wave modulation and to combined stimuli with the flash at 90 and 270°.

### Data analysis

**Fig. 1:**
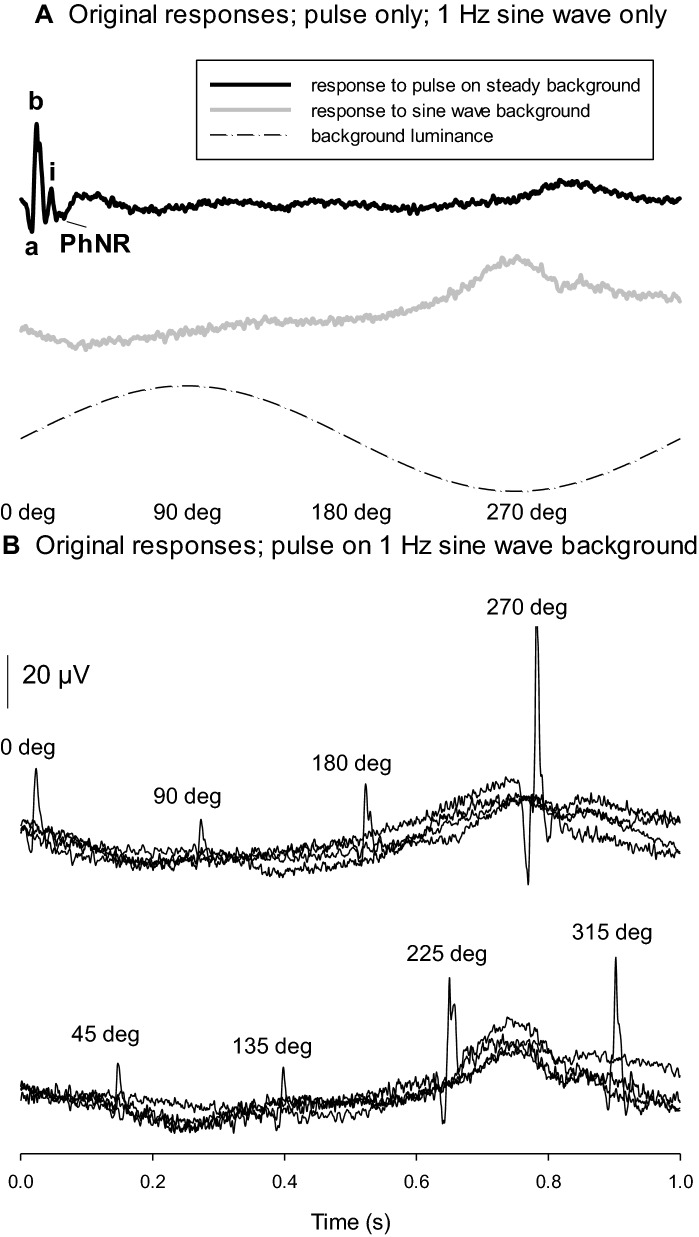
Original responses measured in one observer (SS). **A**: Upper trace: response to a 1 ms 500 cd/m^2^ flash (delivered at *t* = 0 ms) on a 50 cd/m^2^ steady background. The different components that were measured are labeled (see also Fig. 3). Middle trace: response to a 50 cd/m^2^ mean luminance 1 Hz sine wave with 100% contrast. Lower trace: **a** sketch of the luminance of the background. The luminance passes through the mean at *t* = 0 and 0.5 s, it is maximal (twice the mean luminance) at *t* = 0.25 s and 0 cd/m^2^ at *t* = 0.75 s. **B**: Traces of original responses to 1 ms 500 cd/m^2^ flashes upon a 50 cd/m^2^ mean luminance 1 Hz sine wave with 100% contrast. The flashes were delivered at 8 different phases during the sine wave (between 0 and 315° in 45° steps; 0° corresponds to *t* = 0 s, 90° corresponds to *t* = 0.25 s; 180° corresponds to *t* = 0.5 s and 270° corresponds to *t* = 0.75 s; the phases at flash delivery are given above the flash response)

**Fig. 2:**
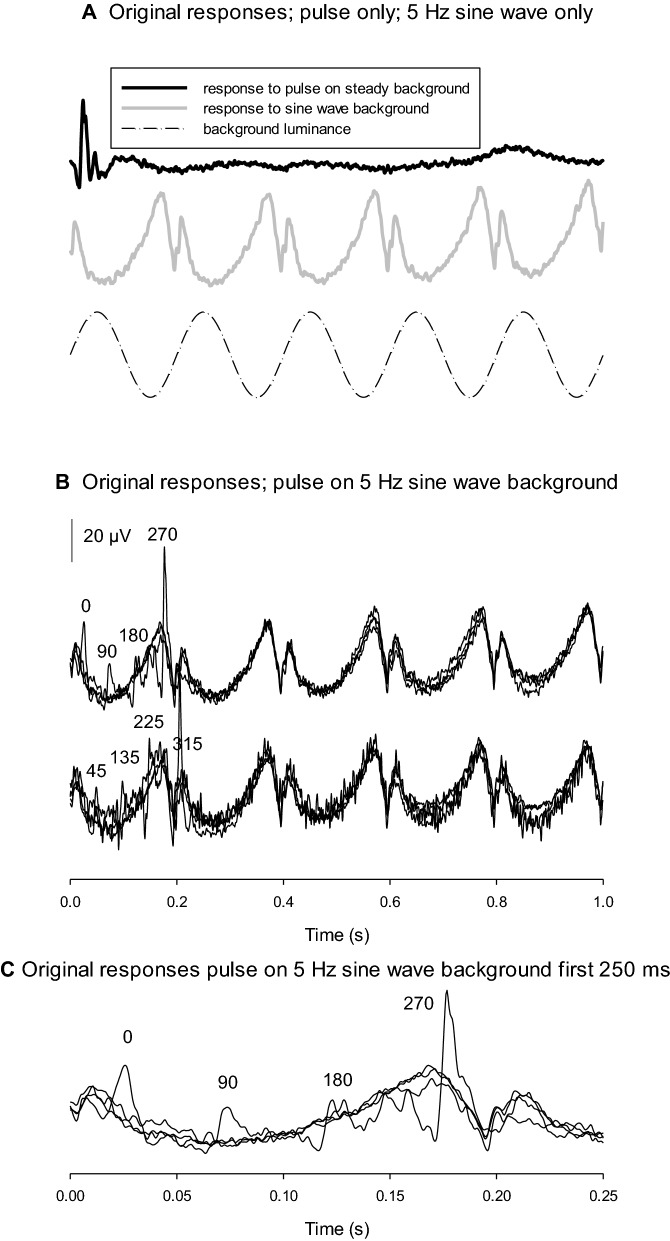
**A** and **B**: Responses in the same observer and the same format as in Fig. 1. The sine wave background was 5 Hz in this case (lower trace in A). Furthermore, the phases corresponded to different times: 0° corresponds to *t* = 0 s, 90° corresponds to *t* = 0.05 s; 180° corresponds to *t* = 0.1 s and 270° corresponds to *t* = 0.15 s. The phases of the flash are given. the responses to the modulating background are complex with a strong negativity (previously called “transient component” [11]). The flash responses at 270 and 315° interfered with this transient component in the response to the sine wave background, which did not alter the morphology of the flash responses. **C**: To show the differences at the differences more clearly, the upper plot of B is replotted with a 250 ms time window

**Fig. 3:**
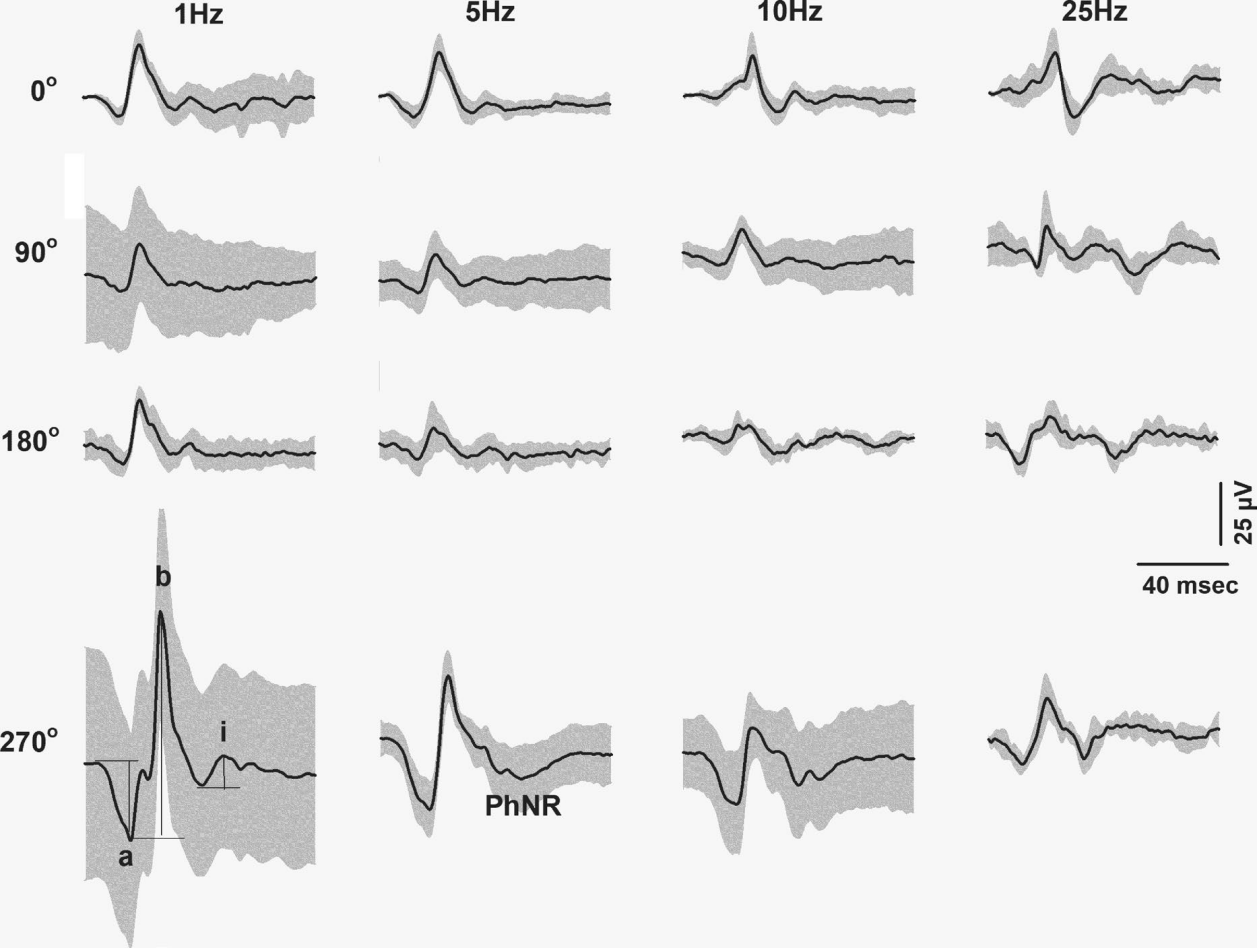
Flash ERGs obtained from the responses to combined flash and a sine wave background after subtraction of the response to the sine wave alone. The drawn curves display the averaged responses and the gray areas are the averages ± 1 SD. DC offsets were reduced offline by adding or subtracting a DC component. The responses are shown for four different phases of flash presentation relative to the sine wave background (given in the different rows). The four columns show the responses for the different frequencies of the modulating background. Reliable results could be obtained from all observers

Figures 1 and 2 show examples of original ERG responses measured in one observer and with 1 and 5 Hz modulating backgrounds, respectively. For each observer, the averaged responses to the sinusoidal stimulus without flash [B] were subtracted from averaged responses to the flash upon the sine-wave background [C] to obtain the responses to the flashes without interference of the ERG responses to the sinusoidally modulating background. We assumed that the flashes (and the ERGs they elicit) do not influence the response to the sine wave. Figures 1B and 2B indicate that this assumption is correct. Figure 3 displays responses after subtraction.

Of all the resulting flash ERGs and of the responses to the flashes upon the steady background, we measured the amplitudes and peak times of two negative and two positive components. The definitions of these components are shown in Fig. 3. The a-wave was measured from baseline (the mean of five data points before the flash) to the minimum in a time window between 19 and 24 ms after flash onset. The b-wave was defined as the maximum between 22 and 34 ms after flash onset minus the minimum of the a-wave. The i-wave was the maximum between 38 and 66 ms post-flash. The amplitude was measured from the preceding minimum. The photopic negative response (PhNR) was defined as minimum following the i-wave and its amplitude was measured relative to the baseline. We also measured the peak times of the different components as the time of the concerning maxima or minima relative to flash onset.

## Results

Figure 1A displays the response to a luminance flash (500 cd/m^2^; 1 ms) on a 50 cd/m^2^ steady background and the ERG elicited by a 1 Hz 100% contrast luminance sine wave in one subject. The latter response may be attenuated because the stimulus and response frequency were close to the high-pass cutoff frequency of the band-pass filter (0.3 Hz) used during the recordings (see methods section). However, we were not interested in the responses to the sine wave stimuli per se but in the influence of the stimulus and/or the response on the flash ERGs (see discussion for arguments that the sine wave itself rather than the ERG response to it influenced the flash ERGs). The lower graph in Fig. 1A shows a sketch of the luminance modulation of the background and the different phases. Figure 1B displays the responses to the 1 ms 500 cd/m^2^ flashes upon the 1 Hz sinusoidally modulating background with flashes presented at different phases during the sine wave background. It can be seen that the flash ERGs strongly depended on the phase of the flash relative to the 1 Hz sine wave background. The responses were small around 90° phase (where the momentary luminance of the modulating background was maximal) and they were maximal at 270° (where the modulating background’s luminance was 0 cd/m^2^). The maximal response as well as those at 225° and 315° were substantially larger than the response upon a steady background. The maximal a- and b-wave was about three to four times larger than the minimal response. Later components, such as the i-wave depended less strongly on the phase. The response to the 1 Hz sine wave background was similar in all conditions. Anecdotally, in some subjects the flashes on a steady background elicited blink artifacts that were absent with flashes on the modulating background, indicating that the flashes with a modulating background were more convenient for these subjects.

Figure 2 displays original responses from the same observer whose responses are shown in Fig. 1 when using a 5 Hz 100% contrast luminance modulating background. In Fig. 2C, the responses to combined stimuli are shown only for the first 250 ms, so that the responses at four phases can be better discerned. Again, the responses to the sine wave background were similar for all conditions. Furthermore, the amplitudes of flash ERGs again depended upon the phase relative to the modulating background. However, close inspection shows two important differences in comparison with the flash ERGs on a 1 Hz background as shown in Fig. 1. First, the amplitude ratio between the minimal and maximal responses was smaller. Second, the phase where the flash ERG was maximal had shifted more closely to 315°.

As mentioned, the ERG responses to the sine wave background was not influenced by the flash or the ERG response to the flash. Therefore, responses to the modulating backgrounds alone were subtracted from the combined responses with the same modulating backgrounds. Figure 3 shows mean flash responses (± 1 SD) at phases 0°, 90°, 180° and 270° after the subtraction. Several features are of interest. First, the dependency of the flash ERG on phase within the modulating background decreased (i.e. the difference between the maximal and minimal response decreased) with increasing background frequency. Second, the responses generally became smaller as background frequency increased, albeit differently for the different components. While a- and b-wave clearly decreased, this was less obvious for the i-wave. Third, with increasing background frequency the maximal response did not occur at 270° and the phase of maximal responses shifted. Fourth, the PhNRs were generally very small and hardly measurable. Finally, with 25 Hz background frequency it was difficult to discern the defined response components because the waveform was variable and depended on the phase.

**Fig. 4:**
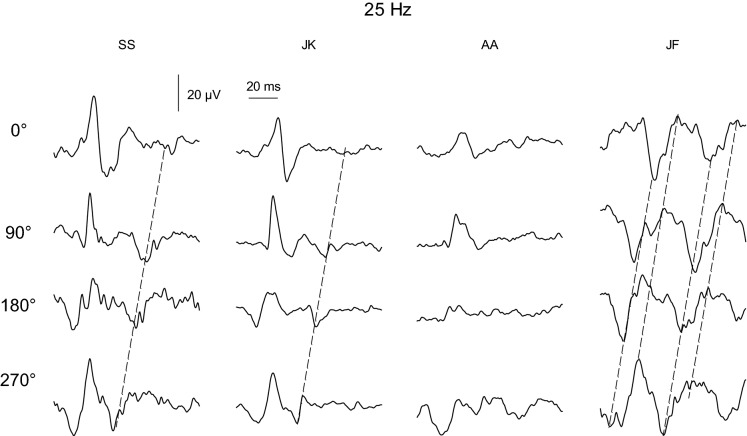
Flash responses with a 25 Hz background stimulus in the four subjects. Conventional flash ERG components were difficult to detect. Furthermore, residual responses to the 25 Hz sine wave stimulus were present, as indicated by the dashed lines that are aligned with the flash time

The variability of the responses with the 25 Hz modulating background is further illustrated in Fig. 4 where the flash ERGs with the 25 Hz background at four relative phases are shown for the four subjects who participated in the measurements with this background. It can be seen that, although the responses vary substantially between different subjects, they display similar features. The responses obtained from subject JF further show that substantial parts of these responses are probably related to the 25 Hz background (because the peaks and troughs are separated by about 40 ms and because the troughs and peaks shift with the phase at which the flashes are delivered, as illustrated by the dashed lines, also observable in the responses of subjects SS and JK), indicating that the subtraction procedure did not completely cancel the responses to the modulating background. It is unclear if this is caused by general variability in the responses to the 25 Hz stimulus or whether the responses to the flashes interfered with those to the 25 Hz background. However, the comparability of the responses in different subjects and different conditions indicates that the effect was systematic rather than stochastic as is expected with random variability. Due to the difficulties in detecting all flash response components and in the interpretation of the responses, they were not considered in further analysis.

**Fig. 5:**
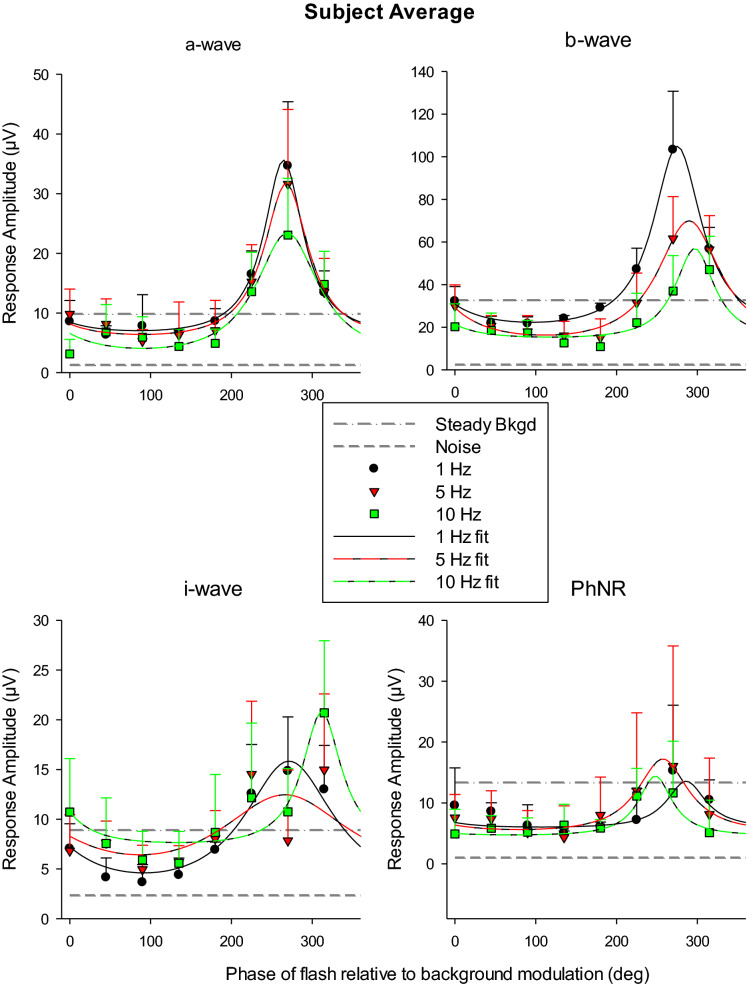
Mean (+ 1 s.d.) amplitudes of the flash ERG components for flashes on a sinusoidally modulating background plotted as a function of the phase relative to the modulating background. The different symbols represent data obtained with different background frequencies. The data are fitted with the function described in the text. The dashed-dotted horizontal line indicates the component’s mean amplitude measured with a steady background. The dashed line represents noise

Figure 5 shows the response amplitudes (means ± 1 s.d.) of the different components as a function of flash phase plotted separately for the 1, 5 and 10 Hz background frequencies. The component amplitudes with the flashes upon a steady background are shown as dashed-dotted horizontal lines. Noise, defined as the result of the same analysis used to extract the different components in the latter part of the recording with the steady background, where no response was present, is given by the dashed horizontal line. Clearly, the amplitudes of the a-, b- and i-waves depended in a systematic manner on phase. These responses were maximal at phases between 270° and 360°. The phase of maximal b-wave responses increased with increasing temporal frequency (although it is difficult to draw a definite conclusion on the basis of the small number of background frequencies). Table 1 gives the ratios of the maximal and the minimal component amplitudes with flashes upon the modulating background relative to those obtained with a steady background. The maximal a-wave amplitude at 1 Hz was about three times the amplitude with flashes on a steady background. The maximal b-wave was about 2.8 times larger than the one with a steady background. The amplitudes of the a- and b-waves decreased as the background frequency increased. The minimal amplitudes were all smaller than the amplitudes in the flash ERGs on a steady background. The influence of the modulating background on the i-wave and the PhNR was less strong. The PhNRs were generally smaller than that of the flash ERG with a steady background. They were also variable.

**Table 1:** Maximal and minimal ratio of the amplitudes of the different components measured with a modulating background relative to their amplitudes on a steady background

Component	Modulating background Frequency (Hz)	Maximal ratio between the component’s amplitudes: with modulating background / steady background	Minimal ratio between the component’s amplitudes: with modulating background / steady background
a-wave	1	3.52	0.64
	5	3.21	0.54
	10	2.34	0.45
b-wave	1	3.16	0.66
	5	1.88	0.46
	10	1.44	0.33
i-wave	1	1.66	0.41
	5	1.68	0.55
	10	2.32	0.62
PhNR	1	1.14	0.37
	5	1.20	0.32
	10	0.87	0.37

As can be seen in Fig. 5, the amplitudes of all components were smaller than those obtained with a steady background for most of the phases. However, the responses were strongly enhanced at phases around 270°, i.e. where the background was 0 cd/m^2^. The PhNR was generally suppressed by the modulating background compared to its amplitude with a steady background. There was no clear dependency on the phase of flash presentation.

The data, shown in Fig. 5, were fitted with functions that were based on the assumptions that the response depended on the momentary Weber fraction (*W*) at phase *P* during the modulating background stimulus: $W\left( P \right) = \frac{{I_{F} }}{{50*sin\left( {P + S} \right)}} = \frac{10}{{sin\left( {P + S} \right)}}$, where *I*_*F*_ is the flash luminance (i.e. 500 cd/m^2^). We further assumed that the physiological processes that produced an internal representation of the background luminance introduced a phase delay (*S*). Because the background luminance is 0 cd/m^2^ at 270°, the corresponding Weber fraction is infinite. If the response *R*(*P*) is defined by the Weber fraction *W*(*P*), a saturating nonlinearity has to be included to ensure that measurable responses are obtained at all phases. We described the saturating nonlinearity by a Naka-Rushton function: $\left( P \right) = c + \frac{{R_{max} *W\left( P \right)}}{b + W\left( P \right)}$ where *R*(*P*) is the ERG response amplitude at phase *P*; *R*_*max*_ is the maximal response, *b* is the Weber fraction for half maximal response and *c* denotes a general (phase independent) noise that is added to the stimulus evoked response. The formula can be rewritten as: $R\left( P \right) = c + \frac{{R_{max} }}{{\frac{b}{W\left( P \right)} + 1}}$. Thus, when *W(p)* becomes infinite then $R\left( P \right) = c + R_{max}$. In the fits there were four free parameters (*R*_*max*_, *b*, *c* and *S*). To constrain the fits, *R*_*max*_ was restricted to values less than twice the maximally measured response. In addition, *b* and *c* were constrained to positive values. The fits were performed separately for the different ERG components and background frequencies and are displayed in Fig. 5. Overall, the fits were satisfactory, indicating that the above model was adequate in describing the ERG response amplitudes. Because there were different numbers of measurements for the different phases (either four or nine), the model was fitted to all individual data (and not to the means). The fits also gave an good description of the mean data.

**Fig. 6:**
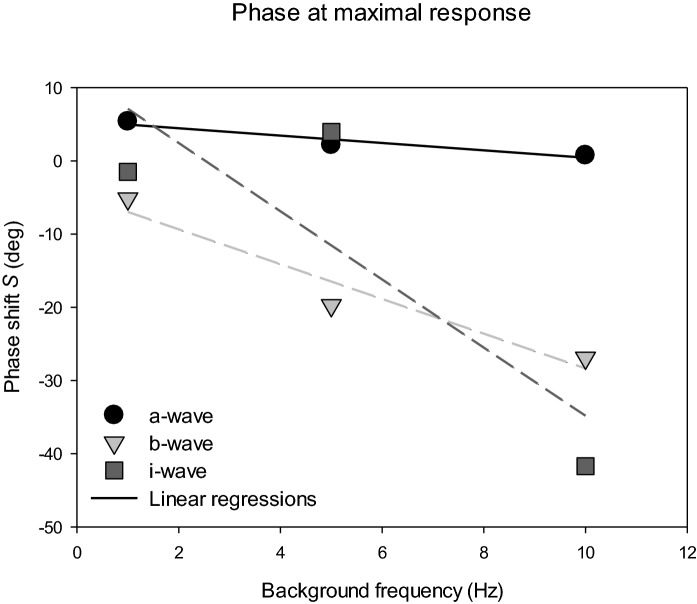
Phase shift *S*, obtained from the fits of the response amplitude vs. modulating background phase data, as a function of the background frequency. *S* represents a delay introduced by internal physiological processing of the background luminance. A linear relationship between the phase shift and background frequency suggests a fixed time delay that determines the slope of the relationship

The values of the parameters *R*_*max*_ and *S* obtained from the model fits are shown in Table 2. By definition, negative values of *S* indicate shifts of the maximal response towards higher phases. (Please observe that the phases are actually modulo of 360°, i.e. integer multiples of 360° can be added or subtracted from these values. We, however, assumed that the phase shifts were delays that were not larger than one cycle of the modulating background period.) *R*_*max*_ for the a-, b- and i-waves decreased with increasing background frequency, indicating increased response suppression or decreased response enhancement. Interestingly, the phase at which the responses were maximal (quantified by *S*) decreased with increasing temporal frequency. The phase shifts are shown in Fig. 6 as a function of the background frequency. Assuming that the phase shift was caused by a fixed time delay in background signal processing, a linear dependency between phase shift and background frequency can be expected with larger delays resulting in steeper slopes. The linear regressions through the data had slopes of 0.50, 2.37 and 4.66°/Hz for the a-, b- and i-waves, respectively. From these slopes, the delays can be calculated by multiplying the slopes with 1000/360 (a phase shift of 360° by a 1 Hz increase in background frequency would indicate a 1 s delay). The delays were estimated to be 1.4 ms for the a-wave, 6.6 ms for the b-wave and 12.9 ms for the i-wave. Measurements at additional background frequencies would result in additional phase shifts and more reliable linear regressions and estimates of the delays.

**Table 2:** Estimates of the parameters *R*_*max*_ and *S* obtained from the model fits to the amplitudes of the different flash response components

Component	Modulating background Frequency (Hz)	*R*_*max*_* (µV)*	*S *(°)
a-wave	1	30.5	5.4
	5	27.5	2.2
	10	22.9	0.8
b-wave	1	91.3	− 5.1
	5	63.6	− 19.7
	10	44.4	− 26.9
i-wave	1	15.8	− 1.5
	5	12.5	4.0
	10	13.8	− 41.7
PhNR	1	8.0	− 10.1
	5	12.9	12.6
	10	10.5	22.8

**Fig. 7:**
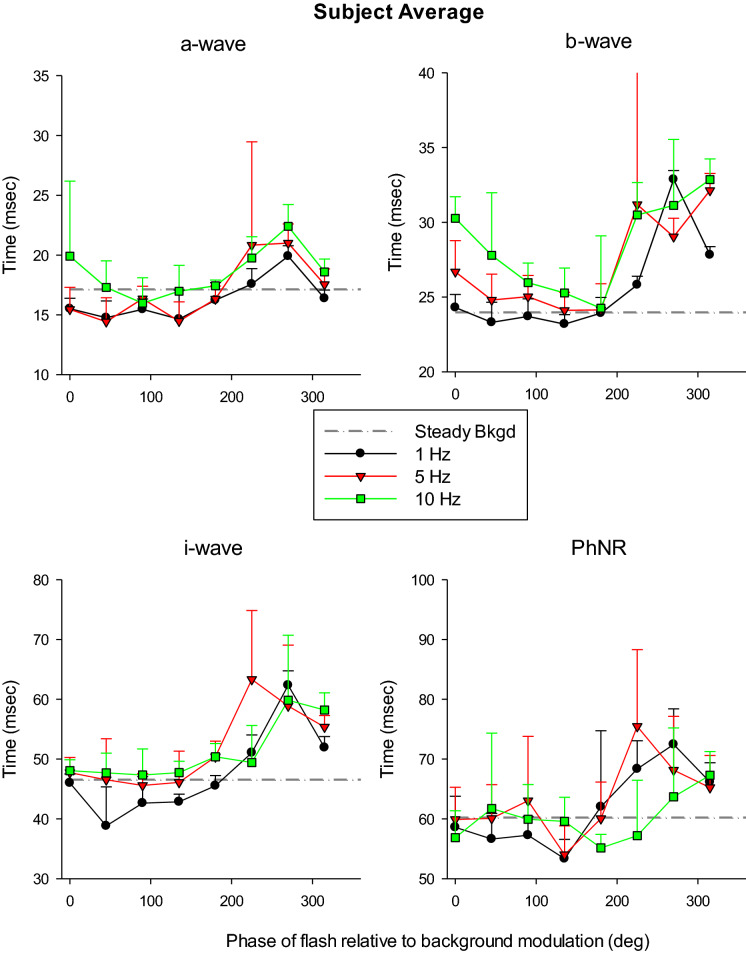
Mean (+ 1 s.d.) peak times of the flash ERG components obtained in measurements with a sinusoidally modulating background given as a function of the phase relative to the modulating background stimulus. The different symbols and lines indicate the date obtained for different background frequencies. The drawn horizontal line indicates the mean flash amplitude on a steady background

**Fig. 8:**
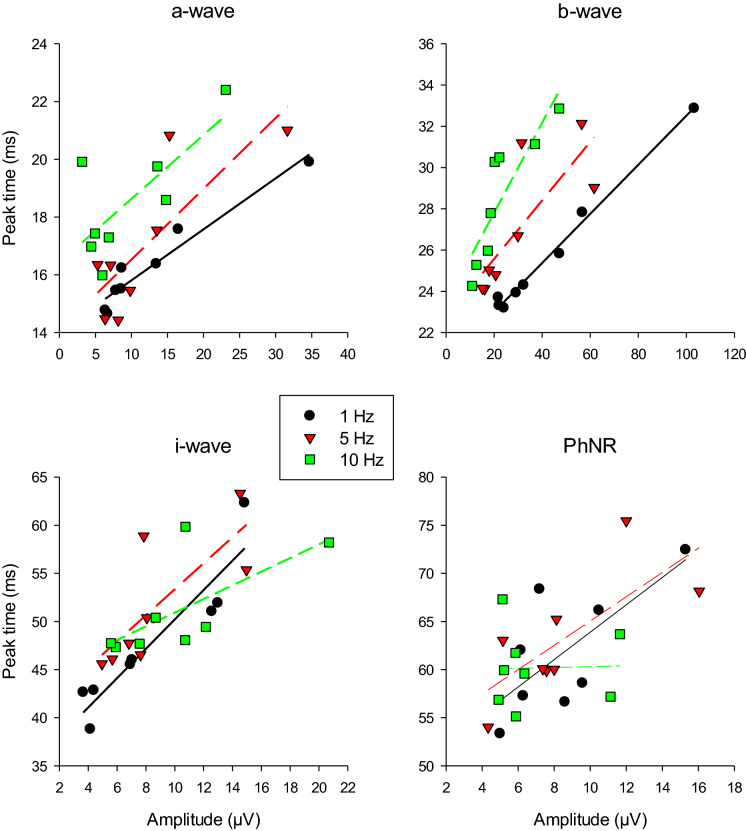
Peak times vs. amplitudes of the different ERG components for the measurements with a modulating background. The different symbols indicate the results for the different modulating background frequencies

Figure 7 displays the peak times (relative to the time of flash onset) of the different components. The peak times of the a-, b-, i-waves and of the PhNRs could vary up to 5, 10, 25 and 20 ms, respectively. In addition, they depended in a systematic manner on the phase relative to the modulating background, and they were maximal at a phase of about 270° where the background was 0 cd/m^2^. Thus, the peak times of the a-, b- and i-waves were large at those relative phases where the responses were also larger and the dependencies of the peak times and amplitudes on relative phase resembled each other. To follow up on this observation, we plotted the peak times as a function of the response amplitudes (Fig. 8) and indeed a correlation could be found for these components and for all frequencies (except at 10 Hz for the PhNR).

For comparison, we measured the responses in three RP patients using a 1 Hz modulating background. The responses to the ISCEV standards showed severely reduced scotopic and photopic ERGs for all three patients.

**Fig. 9:**
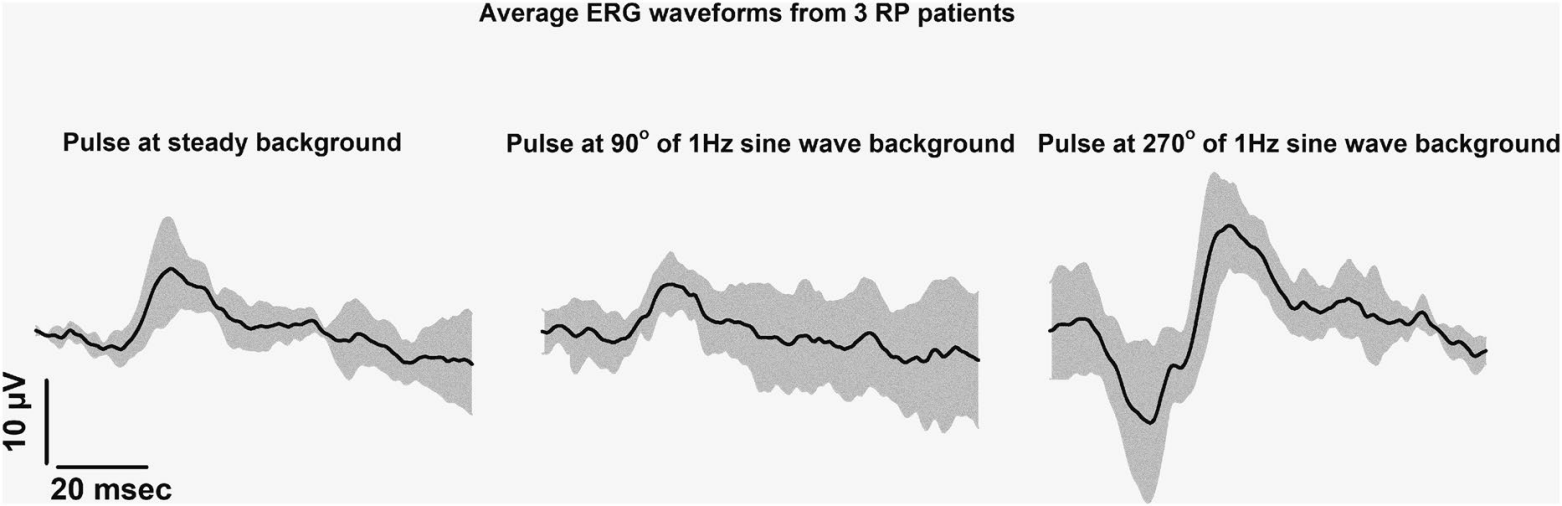
Mean (± 1. s.d) original response of the RP patients to the flash upon a steady background (left trace) and to the flash upon the 1 Hz 100% contrast sine wave modulating background at phase 90° and 270° (middle and right trace respectively)

The left graph in Fig. 9 displays the mean responses (± 1 SD) to the flash upon a steady background. The middle and right graphs display the responses to the combined stimuli with the flash at phases 90° and 270° (see methods) after the subtraction of the response to the sine wave background. Clearly, the responses were reduced compared with those of the normal subjects (cf. the vertical scale bars in Figs. 3 and 9). The response to the flash at 90° phase relative to the sine wave modulating background was reduced relative to the flash upon a steady background, whereas the flash ERG at phase 270° was substantially larger than the other two responses.

**Fig. 10:**
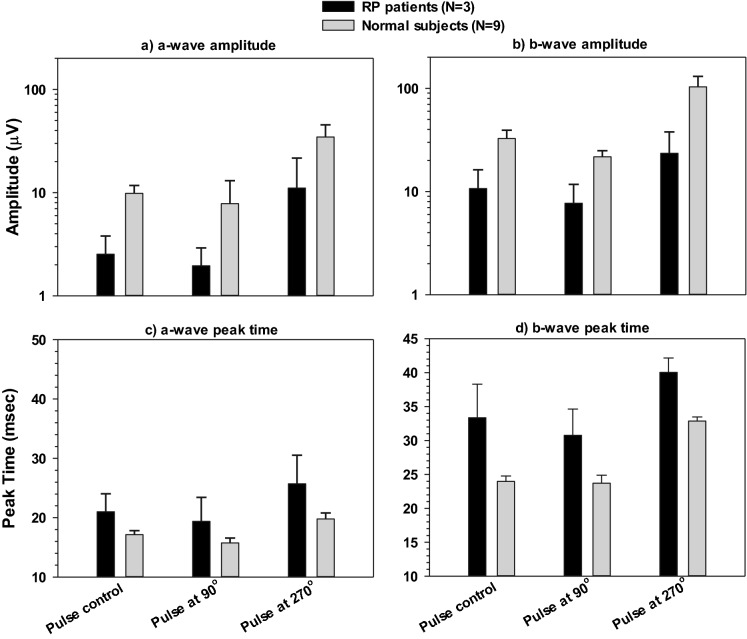
The upper two plots give the mean (+ 1 s.d.) amplitudes of the a-wave (upper left) and the b-wave (upper right) in the RP patients (*n* = 3) and in normal subjects (*n* = 9) measured with 1 Hz modulating background. The lower two plots display the mean (+ 1 s.d.) peak times

In the responses measured with the patients, only the a- and b-waves were clearly visible. We therefore measured their amplitudes and peak times. In Fig. 10, their values are given together with the means (+ 1 s.d.) of those obtained from the normal subjects. The amplitudes of the a-waves in the patient (mean ± s.d. of the amplitudes measured in the normal subjects between brackets) were 2.5 ± 1.2 (9.8 ± 1.8), 1.95 ± 0.96 (7.83 ± 5.23) and 11.09 ± 10.55 (34.65 ± 10.75) µV for the conditions with the steady background, at 90° and at 270° respectively. The b-wave amplitudes were 10.69 ± 5.57 (32.71 ± 6.57), 7.71 ± 4.03 (21.72 ± 3.13) and 23.48 ± 14.31 (103.3 ± 27.41) µV for the conditions with the steady background, at 90° and at 270°, respectively. Observe that the amplitude axes are given logarithmically. The distances between the amplitudes in the normal subjects and the patient are similar for the three conditions, indicating that the responses in the patient were reduced by similar factors.

The lower plots in Fig. 10 display the peak times measured in the patients and the normal subjects. The peak times of the a- and b-waves were substantially larger in the patients.

## Discussion

### Weber fraction as an adequate quantification of flash strength

The goal of the present study was to establish a relationship between flash ERG responses and the momentary Weber fraction of the flash stimuli when using dynamic backgrounds and to characterize ERG mechanisms that process background information. This may give basic insights in signal processing in the retina. Conventionally, static backgrounds are used for the flash ERG measurements. In contrast, in the current study we used sinusoidally modulating backgrounds. The modulations are considered too fast to influence the state of adaptation. All responses to flashes upon the modulating backgrounds had waveforms that were characteristic for a photopic ERG, suggesting that indeed adaptation processes had a negligible influence.

The original responses shown in Fig. 2 (with a 5 Hz background frequency) indicate that the response to the modulating background is complex and deviates from a sinusoid. We previously proposed [11] that high contrast sinusoidal luminance stimuli at frequencies below about 16 Hz elicited complex responses that show two components (which we named “sinusoidal” and “transient”) and with strong negativities leading to the involvement of higher harmonics in frequency domain. The flash ERGs at 270 and 315° phases (Figs. 2 and 3) and their modulations by the sine wave (Fig. 5) do not reflect this complexity and even responses that occur close to the deep negativity in the response to the sine wave (previously called “transient component” [11]) are not affected by it. From this we conclude that the modulation of the flash ERG is caused by processing of the background luminance that is independent of the ERG response to the modulating background. Thus, a model that describes the response modulation as a function of luminance parameters is sufficient. As a consequence, the responses to the sinusoidal modulating background and to the flash are independent and may have different retinal origins. Additional experiments, with more closely spaced phases of the flashes may be necessary to get a clearer picture. The responses to the flashes and to a 25 Hz background were found to be more complex and may interact with each other so that the model may not be adequate at high temporal frequencies.

We found that the flash response amplitudes with modulating background frequencies up to 10 Hz can be very well described with Weber fraction as quantification of the flash strength. However, saturating nonlinearities (described by Naka-Rushton functions) and a delay had to be implemented. Saturating nonlinearities also have to be implemented to describe psychophysical sensitivities [2], ERG responses (e.g. [12]) and responses of single magnocellular retinal ganglion cells [13] to flashed stimuli upon photopic luminance steady backgrounds.

### Modulation of the response amplitudes

The ERG amplitude ratio with a steady and a modulating background (both maximal and minimal amplitudes) generally decreased with increasing frequency for the a- and b-wave (see Table 1). In addition, the values of *R*_*max*_ decreased with increasing frequency for these components (see Table 2). The values for the i-wave and the PhNR did not change as strongly. Furthermore, the maximal ratios were generally smaller for the i-wave and the PhNR. This suggests a fundamental difference in the processing of background luminance in the early compared to the late components. Possibly, the background information is integrated over larger time windows for the later components so that the effect of momentary luminance is diminished. Very slow background modulations would then be expected to have also a large effect on the amplitudes of the late components.

### Internal delays in processing of weber fraction

The model to describe the response amplitudes included a phase shift that was necessary to explain a shift of the relative phase for maximal response with different temporal frequencies of the background. The shift was relatively small for the a-wave, but substantial for the b- and i-waves. Furthermore, the phases  decreased linearly with increasing temporal frequency of the background, suggesting that a fixed delay may be involved. A delay possibly originates in the retinal processes leading to an ERG response. We propose that signals from the background and the flashes, originate at different retinal locations and that a fixed delay time is necessary to interact. The apparent delays increased for the later components and thus with the peak times of the concerning components (although they are not identical because the estimated delays for particularly the b- and i-waves were shorter than their peak times). This indicates that the retinal location of the internal representation of the flash strengths may not be identical with the retinal location where the concerning ERG component originates.

### Relationship between amplitude and peak time

We found a positive correlation between the measured peak times and responses amplitudes for the a-, b-, i-waves and to some extent also for the PhNR. In series with varying flash strengths (intensity response curves) on a fixed steady background, the correlation between the two is negative (although not strong) for the a-wave but positive for the b-wave (e.g. [14, 15]). This suggests that the effect of the background modulation is similar to a modulation of flash strength (at least for the b-wave), which agrees with our suggestion that the Weber fraction determines the response.

### Possible clinical application

We found that the a- and b-waves of the responses to flashes upon a 1 Hz sinusoidally modulating background and presented at the minimum of the sine wave, were up to three times larger than those obtained with a steady background. Although the momentary luminance was about 0 cd/m^2^ the elicited responses were characteristic for a photopic ERG. The measurements with the RP patient show that this amplification can also be found in patients who display decreased ERGs. As a result, the measurements performed in the present study at phases where the background is dim, the comparisons between patient with control data can be studied more reliably. The distinct influences of the modulating backgrounds on the different components make it necessary that normative values should be established again and that they cannot directly be derived from the current normative values for flashes upon a steady background. Possibly, the influence of the background modulation may differ for distinct retinal disorders and thus could be an additional factor that could be used for differential diagnosis.

The flashes recommended by the ISCEV are stronger than those used in the present study. However, we expect that the responses can also be amplified with the stronger flashes. We performed recordings with different flash strengths and found for all stimulus strengths the a-wave was amplified at phase 270°. The b-wave was also amplified and even the largest b-wave amplitude could be further amplified. Interestingly, the photopic hill effect [16] was also increased, so that at the highest flash intensities the amplification disappeared (Aher, Huchzermeyer, Kremers, unpublished data). Furthermore, most subject found that the flashes on a modulating background less inconvenient so that the responses were not often disturbed by blink reflexes.

The amplification was less strong or absent for the i-wave and the PhNR: Assuming that these components originate in the inner retina, this indicates that the method can be particularly interesting for diseases of the outer retina. The i-wave was enhanced with a 10 Hz modulating background, indicating that a higher modulating background frequency might be interesting when this component is of particular interest. More data from more subjects and including more modulating background frequencies are needed to clarify this point.

## Conclusions

The ERG responses to flashes presented upon a sinusoidally modulating background can give additional insights in the physiological processes that are involved. The results indicate that Weber fraction can be used as an adequate quantification of flash strength.

When compared to conventional flash ERGs upon a steady background, the ERG responses with modulating backgrounds are substantially larger for certain phases relative to the sine wave background. This can be used to obtain more reliable characterizations of the diminished responses in patients.

## Declarations

### Statement on the welfare of animals

This article does not contain any research studies with animals performed by any of the authors.
